# Association of polydoctoring and mortality among persons over 85 years with multimorbidity: a prospective cohort study in Japan

**DOI:** 10.3399/BJGPO.2024.0016

**Published:** 2024-09-18

**Authors:** Takayuki Ando, Takashi Sasaki, Yukiko Abe, Yoshinori Nishimoto, Takumi Hirata, Takayuki Tajima, Yuko Oguma, Junji Haruta, Yasumichi Arai

**Affiliations:** 1 Center for General Medicine Education, Keio University School of Medicine, Shinjuku-ku, Japan; 2 Center for Supercentenarian Medical Research, Keio University School of Medicine, Shinjuku-ku, Japan; 3 Department of Neurology, Keio University School of Medicine, Shinjuku-ku, Japan; 4 Human Care Research Team, Tokyo Metropolitan Institute for Geriatrics and Gerontology, Itabashi-ku, Japan; 5 Department of Physical Therapy, Graduate School of Human Health Sciences, Tokyo Metropolitan University, Arakawa-ku, Japan; 6 Sports Medicine Research Center, Keio University, Yokohama, Japan; 7 Medical Education Center, Keio University School of Medicine, Shinjuku-ku, Japan

**Keywords:** care fragmentation, multimorbidity, polydoctoring, aged, cohort studies, primary healthcare

## Abstract

**Background:**

Polydoctoring can increase the risk of care fragmentation among patients with multimorbidity, but its impact on health outcomes remains unclear.

**Aim:**

To determine the effects of polydoctoring, as measured by the regularly visited facilities (RVF) indicator, on patient outcomes among older individuals with multimorbidity.

**Design & setting:**

Data from the ongoing prospective cohort study, Kawasaki Aging and Wellbeing Project (KAWP), was utilised in this study. Among the 1026 KAWP participants aged 85–89 years, those with two or more chronic conditions were enrolled in this study.

**Method:**

Care fragmentation and polydoctoring was evaluated using the RVF, which is a new indicator that measures the number of medical facilities consistently involved in a patient’s care. Based on RVF, mortality was analysed using the Cox proportional hazards model, with adjustments for age, sex, frailty, and number of comorbidities.

**Results:**

A significant reduction in mortality rates was observed in participants with an RVF of ≥3 and 2–4 comorbidities (hazard ratio [HR] 0.43, 95% confidence interval [CI] = 0.18 to 0.99, *P* value = 0.048). However, no significant difference in mortality based on RVF was observed for those with ≥5 comorbidities. Notably, individuals with ≥5 comorbidities and an RVF of 0 had a significantly higher HR for death (HR 2.68, 95% CI = 1.05 to 6.84, *P* value = 0.039).

**Conclusion:**

In older patients with multimorbidity, polydoctoring may reduce mortality in patients with ≤4 coexisting conditions, but it does not significantly impact mortality in those with ≥5 conditions. These findings provide insights for healthcare decision making in managing older patients with multimorbidity.

## How this fits in

While fragmented care is generally considered detrimental, its impact on older people with multimorbidity is inconclusive. The older patients with 2–4 comorbidities treated by multiple specialists demonstrated lower mortality; however, no advantage was conferred to those with ≥5 conditions. Patients without a primary care physician demonstrated higher mortality, emphasising the need for consistent care. Medical services coordination is becoming more important with the ageing of global population, particularly for older patients with multimorbidity.

## Introduction

The increase in older population leads to a rising number of patients with multiple chronic diseases.^1–5^ The escalation of healthcare expenses is influenced by various factors, and it is not conclusively established that ageing is the sole cause of these rising costs.^6^ However, multimorbidity confers health and social-care costs and is considered increasingly significant.^7^ Patients with multimorbidity often require care involving many healthcare professionals, posing a considerable risk of care fragmentation. This care fragmentation refers to inefficient and ineffective medical care resulting from a lack of coordination among healthcare providers.^8^ A systematic review reported that GPs face challenges while managing patients with multiple chronic conditions.^9^ First, referrals to multiple specialists increase the likelihood of fragmented care. Second, neither clear guidelines nor a consensus on best practices for managing patients with multiple comorbid conditions exist, complicating the decision making for GPs.

Previous studies have suggested that care fragmentation can lead to unnecessary tests, hospitalisations, and increased emergency visits.^10–17^ Most studies on care fragmentation are focused on a single disease, such as cancer, stroke, heart disease, or diabetes.^16,18–24^ Some studies have demonstrated that older adults are less likely to have an emergency visit if they are seen by the primary care physician who usually sees them.^11,25^ Contrarily, another study showed that care fragmentation was linked to a lower incidence of hospitalisation among patients with ambulatory care-sensitive conditions.^26^ In a previous systematic review, the impact of fragmentation on mortality in patients with multimorbidity remained inconsistent.^27^ Three studies with older adults were included in this review. One featured multiple continuity-of-care measures; however, the impact of continuous care on mortality proved inconsistent across the patient and claims-based indicators.^28^ This inconsistency in results is possibly due to the differences in the measurement of care fragmentation across studies and the inaccuracy of measures optimally used for multimorbidity.

Many studies have utilised the Fragmentation of Care Index (FCI), which encompasses unplanned visits and reflects emergency condition visits.^29–31^ The consistency of care for acute exacerbations and other unscheduled visits is certainly important, but measures that focus on routine care for chronic conditions are also needed. The situation in which specialists provide regular care for multiple conditions is called polydoctoring and is considered a crucial factor in care fragmentation.^32–35^ Defining 'polydoctoring' only according to the number of involved healthcare facilities might not necessarily reveal fragmented care; although, frequenting multiple facilities significantly contributes to care fragmentation.^35^ Japan’s universal health insurance system guarantees free access to medical facilities to all citizens, allowing self-referral of patients to specialists. Additionally, there are few GP physicians (and considerably more specialists) in Japan; thus, specialists provide general care to many patients, contributing to Japan’s polydoctoring problem.^36^ To address this issue, a novel indicator of polydoctoring called 'regularly visited facilities' (RVF) was developed. This indicator counts the number of medical facilities frequented by patients with multimorbidity.^35^ Our previous research found that higher RVF leads to increased outpatient medical costs.^35^ However, positive or negative impact of high RVF on patient’s health outcomes remains unknown. An improved understanding of these effects will help in guiding resource allocation and better management of patients with multiple comorbidities. The study aimed to elucidate potential associations between patient-specific outcomes and polydoctoring, as measured by RVF.

## Method

### Setting, study population, and data collection

This research utilises data from the ongoing prospective cohort study named the Kawasaki Aging and Wellbeing Project (KAWP). The inclusion criteria of the KAWP were as follows: (1) aged 85–89 years and being a resident of Kawasaki City; (2) no limitations in performing basic activities of daily living (ADLs); and (3) able to visit the study site independently.^35,37,38^ We recruited the cohort between March 2017 and December 2018. The KAWP uses a two-pronged patient data monitoring approach. The first method consists of face-to-face interviews with a multidisciplinary team of professionals, including doctors, nurses, pharmacists, and psychologists along with the collection of physical, psychological, and laboratory data. The second method involves analysis of health and long-term care claims databases and detailing each patient’s healthcare utilisation. Among the 1026 KAWP participants, those with two or more chronic conditions and who consented to have their claims data used were considered for analysis. Two participants did not consent to use of their medical records; 56 others were excluded owing to lack of multiple comorbidities. Finally, 968 patients were included in the study. Information on comorbidities was obtained directly from interviews with physicians and classified into 18 chronic condition groups.^35,38^ Data on outpatient visits were obtained using the health insurance claims database. The observation period was until September 2022, and deaths were confirmed by telephone follow-up and claim data. Missing data on medical history were treated as absent, and other missing values were not imputed.

### Care fragmentation measures

The fragmentation of care is defined as the involvement of multiple medical practitioners without proper coordination.^8^ Care fragmentation is determined by considering the number of healthcare providers involved in a patient’s care and the coordination quality of the care. Given that the quality of coordination is difficult to measure, many studies have analysed the number of medical facility visits.^27^ In patients with multimorbidity, frequent visits to numerous medical facilities are common, referred to as 'polydoctoring'.^35^ Polydoctoring is not synonymous with fragmented care; however, it can increase the risk of its occurrence. Therefore, we have developed the RVF indicator for patients with multimorbidity.^35^ An RVF refers to medical facilities that meet the following two criteria in the health insurance claims data of the subsequent year: (1) having claims data for ≥3 months; and (2) having a gap of ≥6 months between the first and last claims data. These criteria were established because many patients visited their primary care providers thrice yearly. Further, chronic conditions are defined as requiring a minimum of 6 months of prolonged care.^39–42^ RVF is expected to indicate care fragmentation specific to multimorbidity by counting the number of medical facilities involved in the regular care of chronic conditions.^35^ In the present study, the RVF measured over a year after the start of the survey was categorised into 0, 1, 2, and ≥3. Specifically, 0 indicates no regular visits, and 1 indicates visits to only one medical facility. We defined the state where the RVF exceeds 2 as polydoctoring. Polydoctoring is considered a high risk for care fragmentation because an RVF of ≥2 is associated with a higher prevalence of polypharmacy.^35^ Polydoctoring can arise through physician-referral and self-referral practices in Japan. The FCI employed here was determined as done in previous research:^35^


$$FCI\;=\;1\;-\;CCI\;=\;\frac{n^{2}-\;\sum_{i}^{k}n_{i}^{2}}{n\left(n\;-\;1\right)}$$

The FCI is calculated as one minus the Continuity of Care Index (CCI). Here, *n* represents the total number of outpatient visits, *n*_*i*_ represents the number of visits to each facility, *i* and *k* represent the number of facilities visited. Instead of counting each visit day, the number of months with at least one visit to each facility as the number of visits was determined. Based on quartiles, FCI was categorised into four groups. The lowest quartile (Q1) and highest quartile (Q4) comprised patients with the least fragmented and most fragmented care, respectively.

### Other covariates

Age is based on the age at the time of the baseline survey of this cohort study. We included frailty as it is a known risk of all-cause mortality.^43^ Frailty was classified into 'robust', 'pre-frail', and 'frail based on the baseline survey results using the revised Japanese Cardiovascular Health Study (J-CHS) criteria.^44^ The most frequently visited facility (MFVF) was defined as the medical facility with the highest frequency of monthly patient visits.^45^ 'Visit frequency' was determined by counting the months within the claims data where at least one registered encounter occurred. When a patient had the same number of months with at least one visit to a clinic and a hospital, the MFVF was classified as 'both'. Those with no visits were assigned an RVF of 0.

### Outcome measure

The primary outcome was all-cause mortality during the follow-up period. Mortality data were retrieved from the health insurance claims database or by follow-up telephone interviews.

### Statistical analysis

This study is part of extensive investigation related to ageing and wellbeing in the older population. As this was an exploratory project, sample size calculation was not performed. Mortality was treated as a binary event and analysed using the Cox proportional hazards model. Each patient’s RVF was coded as 0, 1, 2, or ≥3. FCI was assessed according to quartiles. Both were incorporated into the model as categorical variables. Adjustments were made for age, sex, and frailty. Frailty was treated as a binary variable (frail versus robust or prefrail) to simplify analysis and reduce overfitting risk in our limited sample. Given the strong correlation between the number of comorbidities and RVF, adjustment using interaction terms was not feasible, so a stratified analysis was performed. The number of comorbidities was categorized into two groups, 2–4 and ≥5, based on the median value within our dataset. Hazard ratio (HR) for death for each RVF was presented. We assessed the validity of our proportional hazards assumption by applying complementary log–log plots. These results indicated that the assumption was upheld, ensuring our analytical approach’s validity. We also conducted a sensitivity analysis limited to the group with the clinic as the MFVF. Statistical significance was set at *α*<0.05. Statistical analysis was conducted using R (version 4.3.1) on RStudio (version 2023.06.1).

## Results

Table 1 displays the characteristics of analysed participants categorised by MFVF. Altogether, 73.9% of the participants had a clinic as their MFVF, 17.8% had a hospital, 2.7% had both, and 5.7% had an RVF of 0. The overall mean (standard deviation [SD]) RVF for the participants was 2.15 (1.30). During the observation period, 158 participants had died, and 22 were lost to follow-up. Table 2 displays patients’ RVF scores associated with their respective number of chronic conditions. Participants with a higher number of comorbidities tended to have higher RVF.

**Table 1.** Description of the characteristics of participants

	By MFVF type				Total
	Clinic	Hospital	Both	None	
Number (%)	715 (73.9)	172 (17.8)	26 (2.7)	55 (5.7)	968
Median age, years (IQR)	86 (85, 88)	86 (85, 87)	86 (86, 88)	87 (85, 88)	86 (85, 88)
Male (%)	375 (52.4)	67 (39.0)	14 (53.8)	31 (56.4)	487 (50.3)
Higher education (%)	322 (45.0)	80 (46.5)	10 (38.5)	24 (43.6)	436 (45.0)
Drinks alcohol (%)	287 (40.1)	71 (41.3)	10 (38.5)	20 (36.4)	388 (40.1)
Smokes (%)	24 (3.4)	6 (3.5)	1 (3.8)	6 (10.9)	37 (3.8)
Independent IADL (%)	617 (86.3)	148 (86.0)	23 (88.5)	47 (85.5)	835 (86.3)
Frailty (%)^a^					
Robust	112 (16.0)	19 (11.3)	5 (19.2)	5 (9.3)	141 (14.9)
Prefrail	417 (59.7)	113 (67.3)	15 (57.7)	29 (53.7)	574 (60.6)
Frail	170 (24.3)	36 (21.4)	6 (23.1)	20 (37.0)	232 (24.5)
Mean number of chronic conditions (SD)	4.75 (1.81)	4.71 (1.71)	5.19 (1.70)	3.69 (1.40)	4.70 (1.78)
Median FCI (IQR)	0.66 (0.52, 0.75)	0.54 (0.29, 0.70)	0.70 (0.65, 0.78)	0.00 (0.00, 0.80)	0.65 (0.48, 0.74)
Mean RVF (SD)	2.39 (1.25)	1.72 (0.88)	3.08 (1.38)	0.00 (0.00)	2.15 (1.30)
0	0 (0.0)	0 (0.0)	0 (0.0)	55 (100.0)	55 (5.7)
1	193 (27.0)	88 (51.2)	0 (0.0)	0 (0.0)	281 (29.0)
2	228 (31.9)	54 (31.4)	10 (38.5)	0 (0.0)	292 (30.2)
≥3	294 (41.1)	30 (17.4)	16 (61.5)	0 (0.0)	340 (35.1)
Number of deaths (%)	105 (14.7)	32 (18.6)	6 (23.1)	15 (27.3)	158 (16.3)

FCI = Fragmentation of Care Index. IADL = instrumental activity of daily living. IQR = interquartile range. MFVF = most frequently visited facility. RVF = regularly visited facilities. SD = standard deviation

^a^Frailty percentages are calculated based on the following denominators: Clinic (*n* = 699), Hospital (*n* = 168), Both (*n* = 54), and Total (*n* = 947) due to missing data on frailty.

**Table 2.** Cross-tabulation of regularly visited facilities (RVF) and the number of chronic comorbid conditions identified in study participants

Chronic conditions	2–4 (*n* = 463)	≥5 (*n* = 505)
RVF		
0	38	17
1	175	106
2	133	159
≥3	117	223

RVF = regularly visited facilities

**Figure 1.**
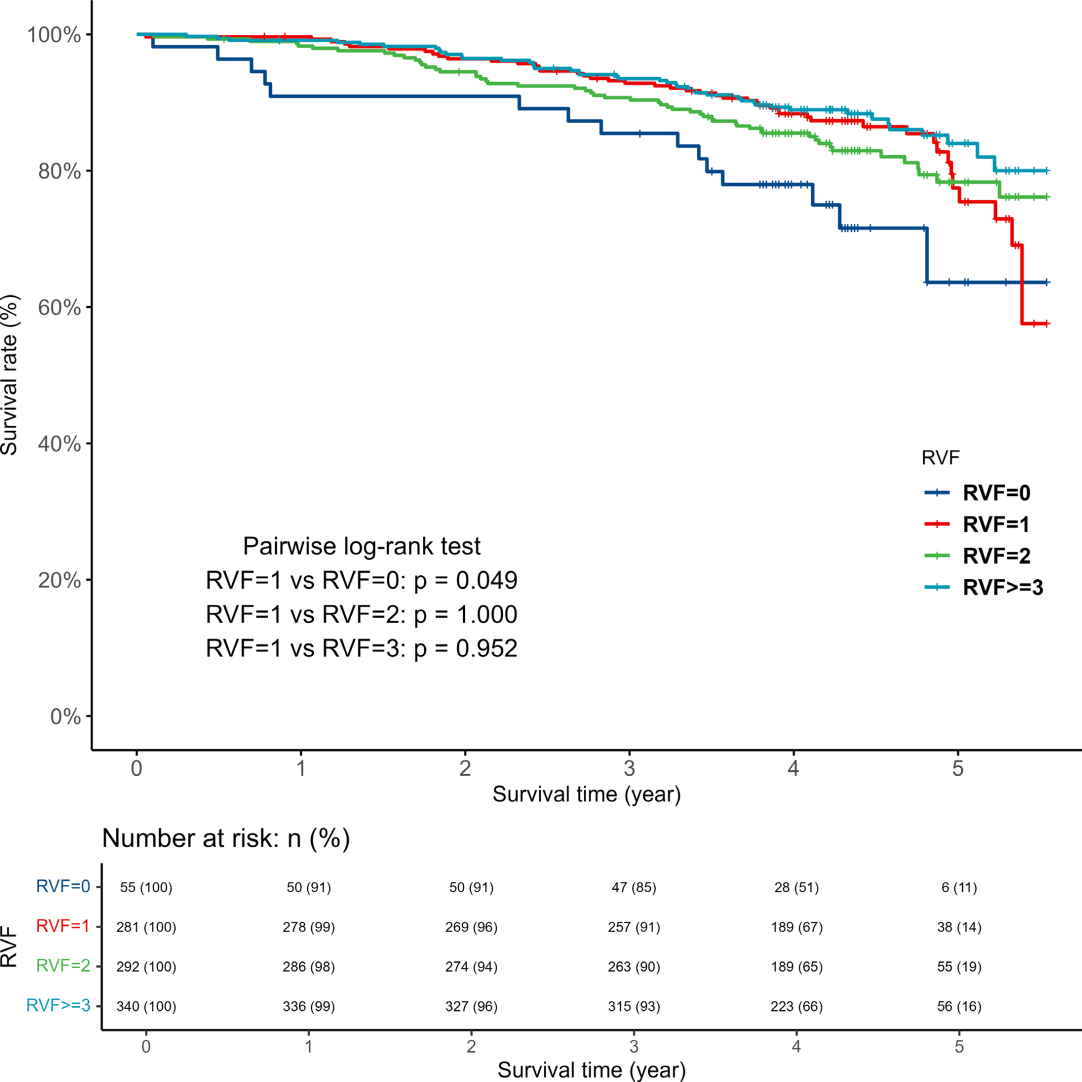
Kaplan–Meier estimates of all-cause mortality according to RVF. RVF = regularly visited facilities

**Figure 2.**
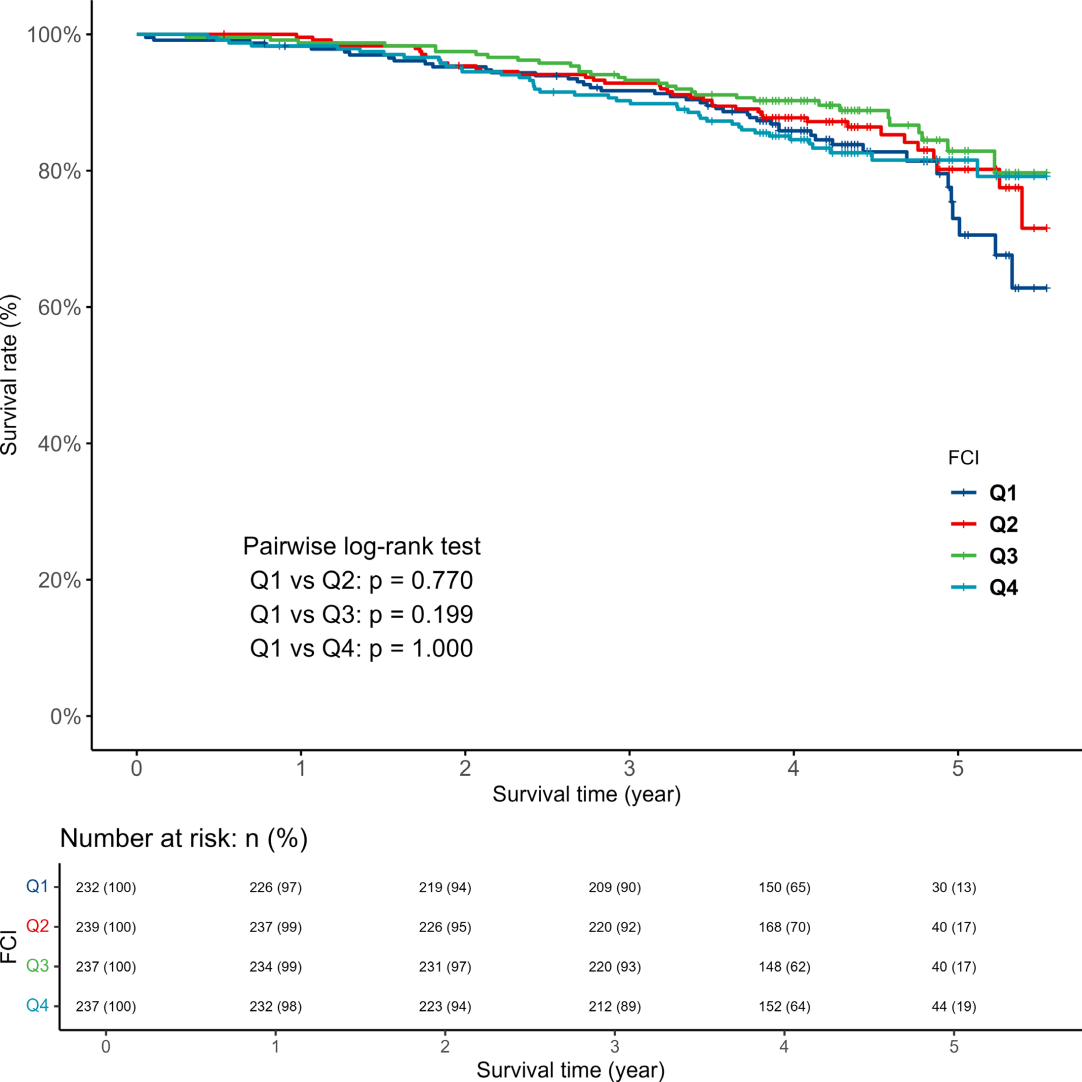
Kaplan–Meier estimates of all-cause mortality, according to the Fragmentation of Care Index (FCI). The 'Q' in Q1, Q2, Q3, and Q4 represents the 'quartile', indicating the first through fourth FCI quartiles.

Figures 1 and 2 depict each RVF and FCI’s Kaplan–Meier curves. The log-rank test indicated a significantly lower survival rate in the RVF 0 group compared with RVF 1 group (*P* value = 0.049). However, there were no significant differences in survival rates between the RVF 1 group and those with RVF values of 2 or ≥3. Compared with the first quartile (Q1) of FCI, which exhibits the least fragmented care, no significant differences in mortality rates were observed in Q2–Q4 patients, as assessed by the log-rank test.

Table 3 displays the HRs for all-cause deaths stratified by the number of comorbidity groups for each RVF. For those with 2–4 comorbidities, the RVF ≥3 group had a significantly lower HR for death than the RVF 1 group (hazard ratio [HR], 0.43, 95% confidence interval [CI] = 0.18 to 0.99, *P* value = 0.048). Contrarily, for those with ≥5 comorbidities, no significant difference in HR was observed, regardless of the RVF. However, in the group with a higher number of comorbidities, an RVF of 0, indicating no regular visit, had a significantly higher HR for death (HR 2.68, 95% CI = 1.05 to 6.84, *P* value = 0.039). In the FCI analysis, the mortality rate was lower in the group with FCI of Q3 in the group with 2–4 comorbidities (HR 0.45, 95% CI = 0.21 to 0.96, P-value = 0.040). Otherwise, FCI was not associated with mortality. Sex and age were not significantly associated with the hazard of mortality. Frailty was associated with an increased hazard of mortality only in the group with severe multimorbidity (≥5 chronic conditions).

**Table 3.** Adjusted associations between regularly visited facilities (RVF) and all-cause mortality

Chronic conditions	2–4 (*n* = 463)		≥5 (*n* = 505)	
	HR (95% CI)	*P* value	HR (95% CI)	*P* value
RVF				
0	1.97 (0.91 to 4.27)	0.085	2.68 (1.05 to 6.84)	0.039
1	Reference	–	Reference	–
2	1.51 (0.86 to 2.64)	0.148	0.84 (0.46 to 1.55)	0.578
≥3	0.43 (0.18 to 0.99)	0.048	0.92 (0.52 to 1.63)	0.774
Sex (male)	0.91 (0.56 to 1.47)	0.687	0.70 (0.45 to 1.08)	0.107
Age (per 1 year)	0.97 (0.82 to 1.16)	0.771	1.05 (0.90 to 1.22)	0.533
Frailty (yes)	0.98 (0.55 to 1.75)	0.950	2.20 (1.43 to 3.39)	<0.001
FCI				
Q1	Reference	–	Reference	–
Q2	0.90 (0.49 to 1.65)	0.739	0.68 (0.35 to 1.33)	0.257
Q3	0.45 (0.21 to 0.96)	0.040	0.85 (0.45 to 1.62)	0.623
Q4	0.84 (0.43 to 1.66)	0.621	0.92 (0.50 to 1.69)	0.779
Sex (male)	0.92 (0.57 to 1.51)	0.750	0.77 (0.50 to 1.20)	0.245
Age (per 1 year)	1.00 (0.84 to 1.19)	1.000	1.06 (0.90 to 1.24)	0.488
Frailty (yes)	1.00 (0.56 to 1.78)	0.994	2.22 (1.43 to 3.45)	<0.001

Multivariate Cox proportional hazard analysis results adjusted for sex, age, and frailty. Participants were stratified into two groups by the median number of comorbid chronic conditions. FCI = Fragmentation of Care Index. HR = hazard ratio. Q = quartile. RVF = regularly visited facilities

Supplementary Table S1 presents the analysis results limited to the group with the clinic as the MFVF. The results remained consistent, that is, no significant association was observed between RVF and the hazard of death for those with ≥5 comorbidities. An association between FCI and mortality was not observed.

## Discussion

### Summary

Polydoctoring was associated with reduced mortality rates in older individuals with a moderate degree of multimorbidity, such as 2–4 comorbidities, highlighting its potential benefits. However, no difference in mortality was observed in those with ≥5 chronic conditions, suggesting a complex interplay between the number of comorbid conditions and the benefits of polydoctoring. Therefore, the number of comorbid conditions should be considered while contemplating specialist referrals. Similar results were obtained in a sensitivity analysis that excluded patients with the hospital as their MFVF; thus, the number of medical facilities may have more influence on patient outcomes than the type of medical facility attended. In the group with severe multimorbidity, the mortality rate was higher in individuals with an RVF of 0 (that is, those with no GP physician). Regular engagement with healthcare providers is crucial for those patients with multiple comorbidities who require continuous care.

In this study, FCI was not associated with mortality, although FCI and RVF were previously found to be correlated.^35^ This may be because FCI and RVF measure different facets of fragmented care. For example, FCI includes unscheduled visits owing to acute illness, whereas RVF focuses on care for chronic illness. Our results suggest that RVF is a reliable indicator of care fragmentation for patients with multimorbidity.

### Strengths and limitations

A key strength of the study is the significant inclusion of a relatively large number of independent older individuals aged ≥85 years. Our findings are particularly relevant for GPs working in outpatient settings, which is an often underrepresented demographic in medical research.

This study has several limitations. First, this is an observational study, suggesting the potential differences in the background characteristics between groups with high and low RVFs. For instance, more active individuals who find it easier to visit other medical facilities might exhibit higher RVFs. Conversely, those with more severe conditions or worse health might visit specialists more frequently, increasing the RVF.^46,47^ However, the study focused on patients who were independent with their ADLs and could visit the study site. These observations suggest that ADLs are not a substantial barrier to referral to other healthcare facilities. Moreover, adjustments were made for frailty, a strong potential confounder.

Second, the study might have an inherent selection bias, as we targeted individuals of above-average socioeconomic status living in mostly urban areas and capable of providing voluntary consent for study participation. Additionally, as the study focused on individuals aged 85–90 years, the results may not be generalised to patients with multimorbidity in other age groups. Hence, future research with larger databases covering a broader population is warranted.

Third, the adjusted comorbidities were simply counted. The sample size was insufficient for distinguishing among specific comorbidity types, severities, or combinations of comorbidities. In the future, studies with large datasets may allow such stratification and potentially elucidate the effects of specific types or combinations of comorbidities on patient outcomes.

### Comparison with existing literature

This study elucidated two key points which are essential for understanding the interplay between the presence of multiple comorbidities and polydoctoring: benefits of seeing a specialist, and treatment burden. Referring to a specialist could lead to more effective care and decreased mortality risk for managing a single disease. A systematic review comparing the health outcomes between specialist and generalist care among patients with a single condition showed inconsistent results, but, for some conditions, the outcomes were better with specialist care.^48^ Some studies have also shown that the mortality rates are lower with specialist care than with generalist follow-up alone, such as in patients with diabetes and heart failure.^49–51^ Specialist care for these conditions possibly contributed to the mortality rate reduction with polydoctoring in the moderate multimorbidity group. However, as the number of chronic conditions increases, the treatment burden might exceed the patient’s capacity, possibly nullifying the benefits of specialist care. This could explain why the mortality difference based on polydoctoring was not observed in those with ≥5 comorbidities. Similarly, the impact of frailty on mortality rates confined to the group with severe multimorbidity can be explained within the treatment burden model framework.^52^ This model acknowledges that complex patients with complicated management plans often create a high treatment burden that might make it difficult to adhere to the prescribed treatments, potentially interfering with efforts to cope with their conditions. An excessive treatment burden may worsen health outcomes while burdening patients and their caregivers. The frail individuals were expected to have lower capacities, and the mortality rate was probably increased in the severe multimorbidity group with a larger treatment burden. Polypharmacy and inappropriate prescriptions for older people could increase the treatment burden. Our previous study indicated that polydoctoring is associated with an increased occurrence of polypharmacy, which is an independent risk factor of mortality.^35,53–55^ Fragmentation of care is also recently reported to be associated with an increased inappropriate prescription among patients with multimorbidity.^56,57^ These prescription issues of polydoctoring could negatively affect patient outcomes and offset the benefits of specialist care.

### Implications for practice

Our study data revealed that, in patients with multimorbidity, polydoctoring reduces the mortality rates in those with ≤4 coexisting conditions. However, it does not affect the mortality risk of those with ≥5 conditions. Given that polydoctoring is associated with higher healthcare costs, care is considered inefficient if it does not reduce the mortality risk of patients with many conditions.^35^ Thus, it is necessary to identify which patient groups would benefit most from specialist referrals and which would not. The results of this study could help primary care physicians make referral decisions for highly independent older individuals aged ≥85 years. Moreover, our study findings can provide valuable insights for designing efficient healthcare systems in Japan and other ageing societies in the era of multimorbidity.

## Data

The dataset relied on in this article is available from the corresponding author on reasonable request.
